# Molecular epidemiology and environmental persistence of methicillin-resistant coagulase-positive *Staphylococci* in a Veterinary Teaching Hospital in Thailand: Evidence for nosocomial transmission and One Health implications

**DOI:** 10.14202/vetworld.2025.3698-3712

**Published:** 2025-12-07

**Authors:** Punpichaya Fungwithaya, Jayaseelan Murugaiyan, David J.Hampson, Nuvee Prapasarakul

**Affiliations:** 1 Office of Administrative Interdisciplinary Program on Agricultural Technology, School of Agricultural Technology, King Mongkut’s Institute of Technology, Ladkrabang, Bangkok 10520, Thailand; 2 Institute for Animal Hygiene and Environmental Health, Faculty of Veterinary Medicine, Freie Universität Berlin, Berlin 14163, Germany; 3 Department of Biological Sciences, School of Engineering and Sciences (SEAS), SRM University-AP, Amaravati, Andhra Pradesh - 522 240, India; 4 School of Veterinary Medicine, Murdoch University, Perth, Western Australia, 6150, Australia; 5 Center of Excellence in Diagnosis and Monitoring of Animal Pathogens, Chulalongkorn University, Pathum Wan, Bangkok 10330, Thailand; 6 Department of Microbiology, Faculty of Veterinary Science, Chulalongkorn University, Pathum Wan, Bangkok 10330 Thailand

**Keywords:** coagulase-positive staphylococci, methicillin-resistant *Staphylococcus pseudintermedius*, nosocomial infection, One Health, *staphylococcal cassette mec* typing, veterinary hospital

## Abstract

**Background and Aim:**

Methicillin-resistant coagulase-positive *Staphylococci* (MRCoPS), including methicillin-resistant *Staphylococcus pseudintermedius* (MRSP), methicillin-resistant *Staphylococcus aureus* (MRSA), and methicillin-resistant *Staphylococcus coagulans* (MRSC), are emerging zoonotic pathogens in veterinary hospitals, posing significant infection control challenges. This study aimed to investigate the molecular epidemiology, antimicrobial resistance profiles, and clonal dissemination of MRCoPS across environmental surfaces, veterinary personnel, and canine patients at the Veterinary Teaching Hospital, Chulalongkorn University (VTH-CU), Thailand.

**Materials and Methods:**

A cross-sectional study was conducted involving 216 environmental samples, 23 veterinary staff, and 14 canine patients. Isolates were identified using biochemical tests, polymerase chain reaction (PCR), and matrix-assisted laser desorption/ionization–time-of-flight mass spectrometry. Methicillin resistance was confirmed by *mecA* gene detection. Antimicrobial susceptibility was evaluated through disk diffusion following Clinical and Laboratory Standards Institute guidelines. Molecular typing was performed using *staphylococcal cassette mec* (SCC*mec*) PCR and pulsed-field gel electrophoresis (PFGE). Multivariate logistic regression identified environmental predictors of MRCoPS contamination.

**Results:**

Among 88 coagulase-positive isolates, 62 (70.5%) were methicillin resistant, predominantly MRSP (91.9%), followed by MRSC (8.1%) and MRSA (1.6%). Floors represented the principal environmental reservoir, with significantly higher contamination odds than medical instruments (adjusted odds ratios [AOR] = 0.32; 95% confidence interval = 0.12–0.86; p = 0.024). The dermatological unit showed a six-fold higher risk of MRCoPS contamination than the medicine unit (AOR = 5.86; p = 0.027). All MRSC isolates carried SCC*mec* type V and displayed a consistent triple resistance pattern (gentamicin-clindamycin-erythromycin), while MRSP isolates exhibited diverse antibiograms and untypeable SCC*mec* elements. PFGE revealed clonal similarity (pattern A) between canine and environmental isolates, confirming the potential for nosocomial transmission.

**Conclusion:**

MRCoPS, particularly MRSP, were widely distributed and persistent in the VTH-CU environment, despite routine cleaning. The clonal overlap among environmental and canine isolates highlights potential cross-contamination within the hospital. Strengthened disinfection protocols, antimicrobial stewardship programs, and regular environmental surveillance are imperative to mitigate the spread of multidrug-resistant staphylococci. This study highlights the importance of integrating environmental, animal, and human infection control practices in veterinary facilities within the context of One Health.

## INTRODUCTION

Coagulase-positive *Staphylococci* (CoPS) are Gram-positive cocci responsible for a variety of infections, including dermatitis, wound infections, and septicemia in animals and humans [1–3]. Among these, *Staphylococcus aureus*, *Staphylococcus pseudintermedius*, and *Staphylococcus coagulans* are the most clinically relevant species that colonize and cause disease in animals [4–7]. Importantly, canine CoPS can exchange the mobile genetic element known as the staphylococcal cassette chromosome *mec* (SCC*mec*) both within and between species [8–10]. This element carries multiple antibiotic resistance determinants, particularly the *mecA* gene, which encodes resistance to β-lactam antibiotics such as oxacillin.

Methicillin-resistant CoPS (MRCoPS) harboring the *mecA* gene represent a major concern in both human and veterinary healthcare environments due to their multidrug-resistant (MDR) nature and zoonotic potential. These pathogens can be transmitted among humans, animals, and contaminated surfaces, including medical instruments [11–13]. Although methicillin-resistant *S. aureus* (MRSA) is typically isolated in low numbers from veterinary hospitals and small-animal skin, it can occasionally cause severe outbreaks, as documented in equine facilities [14]. In humans, MRSA is associated with localized and systemic infections, including septicemia [15, 16]. In contrast, methicillin-resistant *S. pseudintermedius* (MRSP) commonly exhibits MDR to β-lactams, macrolides, lincosamides, fluoroquinolones, and aminoglycosides [13, 17, 18]. The growing prevalence of MRSP in veterinary hospitals has heightened the risk of surgical site infections and septicemia in companion animals, posing significant challenges to infection control and hospital management [19, 20]. Consequently, MRSP has emerged as a leading cause of nosocomial infections in veterinary settings [21].

Environmental surfaces, veterinary staff, and animal patients serve as critical reservoirs and vectors for MRSA, MRSP, and methicillin-resistant *S. coagulans* (MRSC) within clinical environments [22]. Continuous monitoring of MRCoPS is therefore essential to predict and mitigate their spread [22, 23]. These organisms have been recovered from diverse surfaces and instruments, such as floors, examination tables, stethoscopes, and weighing scales, indicating widespread environmental persistence [13, 22, 24]. The persistence of MRCoPS even after standard disinfection underscores the risk of ongoing cross-contamination [18, 23]. Transmission can also occur between pets, veterinarians, and owners, facilitated by inadequate hygiene and contaminated medical devices. Notably, the prevalence and distribution of MRCoPS vary across hospitals, depending on disinfection practices, hand hygiene, and infection control standards [25, 26]. Thus, rigorous hygiene policies and accreditation protocols, including adherence to proper hand hygiene, use of protective clothing, and application of effective disinfectants, are imperative for controlling MRCoPS dissemination [25].

In Thailand, research on MRCoPS in veterinary hospitals has primarily focused on MRSA, with limited attention to other coagulase-positive species. Early investigations characterized MRSA in humans, pets, and the hospital environment but did not extend to MRSP or MRSC [27]. Our previous study was among the few to explore transmission dynamics involving staff, animal patients, and the surgical unit environment, providing critical insight into potential pathways of hospital contamination [19].

Despite growing awareness of methicillin-resistant Staphylococcus species in veterinary hospitals, existing literature has several critical limitations. Most prior investigations in Thailand have centered on *S. aureus* (MRSA), often overlooking the broader spectrum of CoPS (MRCoPS), particularly *S. pseudintermedius* (MRSP) and *S. coagulans* (MRSC), which are increasingly recognized as major nosocomial and zoonotic pathogens. Moreover, previous environmental surveillance studies have largely been descriptive, focusing on detection frequency rather than exploring the molecular epidemiology and clonal relationships among isolates from animals, humans, and hospital surfaces. Consequently, there remains a limited understanding of how MRCoPS persist in different clinical units, their antimicrobial resistance mechanisms, and their genetic relatedness, all of which are essential for determining infection sources and transmission routes. In addition, the efficacy of disinfection and hospital-specific risk factors influencing the persistence of these organisms have rarely been statistically evaluated. The lack of integrative, cross-sectional molecular studies hampers the development of evidence-based infection control strategies in veterinary settings, particularly in tropical hospital environments where cleaning conditions, antibiotic usage, and patient load may differ significantly from those in temperate regions.

This study aimed to investigate the molecular epidemiology, antimicrobial resistance patterns, and environmental distribution of MRCoPS in a major veterinary teaching hospital (VTH) in Thailand. Specifically, the study sought to (i) determine the prevalence and diversity of MRCoPS species recovered from environmental surfaces, veterinary personnel, and canine patients; (ii) identify *mec*A-positive isolates and characterize their SCC*mec* types; (iii) evaluate antimicrobial susceptibility profiles to commonly used veterinary antibiotics; (iv) determine the clonal relationships among isolates using pulsed-field gel electrophoresis (PFGE); and (v) analyze environmental and operational risk factors contributing to MRCoPS persistence using multivariate logistic regression. By integrating microbiological, molecular, and epidemiological data, this research provides the first comprehensive evidence of MRCoPS transmission dynamics within a Thai veterinary hospital context, supporting the design of targeted infection control and antimicrobial stewardship strategies aligned with the One Health framework.

## MATERIALS AND METHODS

### Ethical approval

**Figure 1:**
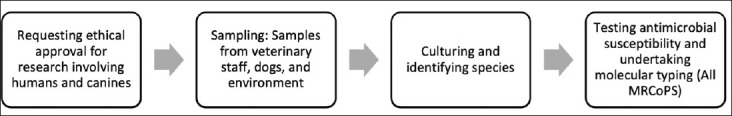
Outline of the study workflow.

Ethical clearance for the study was obtained from both the Institutional Animal Care and Use Committee (approval no. 113/56) and the Ethical Review Committee for Research Involving Human Research Subjects, Health Science Group, Chulalongkorn University (approval no. 137/57), Research and Innovation for Society, Chulalongkorn University. The overall workflow of the study is summarized in Figure 1.

### Study period and location

A cross-sectional study was conducted during May 2014 at the VTH, Chulalongkorn University (VTH-CU), within the Small Animal Teaching Hospital of the Faculty of Veterinary Science, Bangkok, Thailand.

### Hospital setting

The VTH-CU is one of the largest veterinary referral hospitals in Thailand, handling approximately 140,000 patient visits annually. The hospital comprises five main divisions: Intensive Care Unit (ICU), General Medicine, Surgical, Gynecological, and Special Units, including Cardiology, Nephrology, Diabetes, Dermatology, Oncology, Feline Medicine, and Neurology. During the study period, an average of 389 animal patients visited the hospital daily, distributed across General Medicine (96 patients), Gynecology (30), Dermatology (27), and Surgery (8).

The hospital’s sanitation management routine was standardized but varied slightly by location. Hallways were mopped daily using a 2.5% (w/v) quaternary ammonium compound disinfectant (Umonium38®; Laboratoire Huckert’s International, Thailand) between 3:30 PM and 4:00 PM. Examination tables, stethoscopes, syringe trays, waiting benches, drug cabinets, keyboards, and doorknobs were sanitized with 0.5% (w/v) Umonium38® spray during off-patient hours. Sampling locations were selected based on previously identified high hand-touch areas [28]. Wound-dressing cotton was stored in the same examination room until use. Forceps jars containing 1% (w/v) povidone–iodine solution (Betadine®; Pathum Thani, Thailand) were replenished daily.

### Sampling strategy

#### Environmental sampling

A total of 216 environmental samples were collected from nine functional units of the VTH-CU: ICU (C), Dermatological Unit (SW), Gynecology (G), General Medicine (M), Post-surgery Care (P), Surgery (S), Vaccination (V), and both lower and upper hallways (L, U). At the time of collection, the Medicine, Dermatological, and Vaccination units were located in a newly constructed building, while Gynecology, Post-surgery, Surgery, and hallway areas were part of the older hospital wing.

The room inclusion criteria required a minimum of 8 animal patients per day. Environmental samples were collected twice on the same weekday, before clinic opening (7:30–8:00 AM) and after daily cleaning (3:30–4:00 PM). For each room, swabs were taken from five specific floor areas and frequently touched instruments, as summarized in Table 1.

**Table 1:** Criteria used for sample collection from floors and medical equipment and fittings.

Environmental samples	Type	Sample/area/time	Swabbing criteria (area per swab)
Floor	Floor	5/1/1	3 × 3 cm^2^ from five parts: right up, right down, middle, left up, and left down in the main examination room
Medical equipment and fittings	Wound cotton	1/1/1	Bandaging wound cotton (1 g) in the main examination room
	Stethoscope	1/1/1	0.1 × 0.1 cm^2^ of the animal contact surface of the stethoscopes in the main examination room
	Syringe tray	1/1/1	0.1 × 0.1 cm^2^ of syringe tray in the main examination room
	Disinfectant water^1^	1/1/1	1 mL disinfectant water in a forceps jar in the main examination room
	Examination table	1/1/1	10 × 10 cm^2^ on the examination table surfaces in the main examination room
	Waiting bench	1/1/1	10 × 10 cm^2^ waiting bench in front of the main examination rooms
	Drug cabinet	1/1/1	10 × 10 cm^2^ of drug cabinet in the division or unit in the main examination room
	Keyboard	1/1/1	10 × 10 cm^2^ of keyboard in the main examination room
	Doorknob	1/1/1	0.1 × 0.1 cm^2^ of doorknob on the front door in the main examination

Negative control: sterile water containing 1% (w/v) povidone-iodine (Betadine solution; Pathum Thani, Thailand).

Sampling was performed using sterile cotton swabs pre-moistened with 2 mL of peptone-saline diluent (100 mg/mL peptone and 850 mg/mL sodium chloride). The swab was rolled over the defined surface area, after which the cotton tip was aseptically detached, placed into the same tube, stored on ice, and processed within 2 h.

#### Veterinary staff sampling

One veterinary nurse from each clinical unit and 16 veterinarians, each with more than 2 years of service and over 40 working h/week, were enrolled. All participants routinely used protective masks while on duty. Sterile swabs were used to collect nasal cavity samples according to the standard protocol described by Chanchaithong *et al*. [29].

#### Canine patient sampling

Fourteen samples were obtained from superficial wound abscesses of dogs admitted to the Dermatological, Post-surgery Care, and Surgery rooms on the same days as environmental and personnel sampling. Outpatient dogs with wound infections or dermatitis were included following approval from the attending veterinarian. Each sterile swab was moistened in 2 mL of peptone saline diluent and applied to the wound site following the treatment protocol described by Chanchaithong *et al*. [29].

### Culture and identification of isolates

All samples were cultured within 2 h of collection. A 0.1 mL aliquot of each bacterial suspension was inoculated onto Baird–Parker agar (Difco, France) plates with and without 0.5 µg/mL oxacillin for Staphylococcus screening, performed in duplicate. Black, *Staphylococcus*-like colonies were sub-cultured on tryptic soy agar (Difco) and examined for coagulase activity. Up to three coagulase-positive colonies per sample were retained for analysis.

Species identification was performed using standard biochemical tests [30], multiplex polymerase chain reaction (M-PCR), and matrix-assisted laser desorption/ionization–time of flight mass spectrometry (MALDI-TOF MS) [31, 32]. Control strains included *S. aureus* American Type Culture Collection (ATCC) 25923, *S. pseudintermedius* CVMC 0108, *S. coagulans* CVMC 0208, *Staphylococcus intermedius* CVMP 0309, *Staphylococcus delphini* CVMP 0109, and sterile water as the negative control.

Oxacillin (1 µg) and cefoxitin (30 µg) disk diffusion tests were performed according to Clinical and Laboratory Standards Institute (CLSI) guidelines [33]. Methicillin resistance was confirmed by *mec*A gene detection via PCR [34]. *S. aureus* ATCC 25923 and MRSA N315 served as positive controls.

#### Antimicrobial susceptibility testing

All confirmed MRCoPS isolates were tested for antimicrobial susceptibility using the disk diffusion method [33]. Antibiotics included clindamycin (DA) (2 µg), doxycycline (DO) (30 µg), erythromycin (E) (15 µg), gentamicin (CN) (10 µg), mupirocin (200 µg), and sulfamethoxazole/trimethoprim (25 µg) (Oxoid/Thermo Fisher Scientific, UK). *S. aureus* ATCC 25923 served as the control strain. Oxacillin (1 µg) was used for MRCoPS testing, and cefoxitin (30 µg) for *S. aureus*. Results were interpreted according to CLSI (2013) breakpoints; DO breakpoints were determined according to Maaland *et al*. [35].

#### Molecular typing of MRCoPS

The *mec* and *ccr* gene complexes were characterized in all MRCoPS isolates by M-PCR to determine SCC*mec* types [36]. Clonal relatedness was evaluated using PFGE following the method of Chanchaithong *et al*. [29]. Genomic DNA embedded in Seakem® agarose (Bio-Rad, USA) was digested with Cfr9I or ApaI restriction enzymes [37]. DNA fragments were separated using a CHEF-DR III apparatus (Bio-Rad) at 6 V/cm and 14°C with a pulse-switching interval of 5–15 s for 18 h, followed by 15–60 s for 5 h. Gels were stained with Red Safe™ Nucleic Acid Stain (iNtRON Bio, NSW, Australia) and visualized under ultraviolet illumination. A Lambda Ladder PFGE marker (New England BioLabs, USA) served as a size standard. Dendrograms were generated using BioNumerics software version 4.0 (Applied Maths, Kortrijk, Belgium) with the Dice coefficient (1.5%) and Unweighted Pair Group Method with Arithmetic mean (UPGMA) clustering, applying an 80% similarity threshold to define distinct pulsotypes [38].

### Statistical analysis

All statistical analyses were performed using the Statistical Package for the Social Sciences Statistics 22 (IBM Corp., Chicago, IL, USA). The prevalence of Staphylococcus species was expressed as percentages for CoPS, methicillin-resistant (MRCoPS), and methicillin-susceptible CoPS (MSCoPS) isolates.

Associations between environmental factors and MRCoPS occurrence were analyzed using Chi-square or Fisher’s Exact Tests, with 95% confidence intervals (CI). Odds ratios (ORs) and adjusted odds ratios (AORs) were calculated to identify independent risk factors using multivariate logistic regression. Independent variables included room type (Gynecology, Post-surgery, Dermatological; Medicine as the reference), surface type (medical instruments vs. floor), cleaning frequency (once vs. twice daily), and antiseptic use (present vs. absent). Statistical significance was set at p < 0.05.

## RESULTS

### Prevalence and distribution of CoPS

A total of 253 samples were collected from environmental surfaces (n = 216), veterinary personnel (n = 23), and canine patients (n = 14) across nine functional units of the VTH-CU. Among these, 88 CoPS isolates were obtained from 48 positive samples, representing an overall isolation rate of 18.97%. Specifically, 40 isolates originated from environmental surfaces (15.81%), seven from canine wound swabs (2.76%), and one from the nasal cavity of a veterinarian (0.40%) (Table 2).

**Table 2:** Hospital units, main antibiotic in use, routine cleaning management, positive surfaces, odds ratio, 95% confidence interval, and *P*-value of MRCoPS.

Unit	Main antibiotic used	Routine cleaning management (antiseptic, equipment, and time)	Average number of colonies on the floor					Average number of colonies on medical instruments^a^				

			CoPS positive surfaces (%)	MRCoPS-positive surfaces (%)	MRCoPS-positive surfaces			CoPS positive surfaces (%)	MRCoPS-positive surfaces (%)	MRCoPS-positive surfaces		
	
					Odds ratio	95% CI	p-value			Odds ratio	95%CI	p-value
Gynecology	Enrofloxacin	2.5% quaternary ammonium compound/Mob/1	5/10 (50)	5/10 (50)	4	0.50–31.84	0.294	3/18 (16.67)	2/18 (11.11)	2.13	0.17–26.15	1
Medicine	Amoxicillin/clavulanic acid	2.5% quaternary ammonium compound/Mob/1	4/10 (40)	2/10 (20)	Reference^1^	Reference^1^	Reference^1^	3/18 (16.67)	1/18 (5.56)	Reference^1^	Reference^1^	Reference^1^
Post-operative care	Enrofloxacin	2.5% quaternary ammonium compound/Mob/2	3/10 (30)	3/10 (30)	1.71	0.22–13.51	0.76	4/18 (22.22)	3/18 (16.67)	3.4	0.31–37.75	0.551
Surgery	Enrofloxacin	2.5% quaternary ammonium compound/Mob/1						1/18 (5.56)				
Dermatological	Amoxicillin/clavulanic acid, cephalexin, and metronidazole	No antiseptic/ Broom/1	6/10 (60)	6/10 (60)	6	0.79–45.72	0.155	3/18 (16.67)	3/18 (16.67)	3.4	0.31–37.75	0.551
ICU	Amoxicillin/clavulanic acid, cephalexin, and metronidazole	2.5% quaternary ammonium compound/Mob/2	2/10 (20)					2/18 (11.11)	1/18 (5.56)	Reference^1^	Reference^1^	Reference^1^
Vaccine		2.5% quaternary ammonium compound/Mob/1	2/10 (20)									
Lower floor		Unknown/Mob/1	1/10 (10)									
Upper floor		Unknown/Mob/1	1/10 (10)									

a Medical instrument = Wound cotton, stethoscope, disinfectant water, syringe tray, examination table, waiting branch, drug cabinet, keyboard, and doorknob.

1 Reference = The Medicine unit was selected as the reference room for both floor and medical instruments because it displayed the lowest number of MRCoPS in this study. Gray box = Not detectable. MRCoPS = Methicillin-resistant coagulase-positive *Staphylococci*, CI = Confidence interval, ICU = Intensive care unit

Among the 88 CoPS isolates, *Staphylococcus pseudintermedius* was the predominant species (n = 75; 85.23%), followed by *S. coagulans* (n = 12; 13.64%) and a single isolate of *S. aureus* (1.14%) obtained from a veterinary staff member. Methicillin resistance was detected in 62 isolates (70.5% of all CoPS), comprising 57 MRSP (91.9%) and 5 MRSC (8.1%).

### Environmental and operational risk factors for MRCoPS contamination

Multivariate logistic regression analysis was performed to determine environmental predictors associated with MRCoPS contamination. The analysis identified surface type and room type as the major independent factors influencing contamination levels. After adjusting for confounding variables, medical instruments were significantly less likely to harbor MRCoPS compared with floor surfaces (AOR = 0.32; 95% CI: 0.12–0.86; p = 0.024). Floors were therefore identified as the principal environmental reservoirs for MRCoPS in the hospital.

The dermatological unit demonstrated the highest contamination risk, with odds of MRCoPS detection approximately 6 times greater than those in the medicine unit (AOR = 5.86; 95% CI: 1.22–28.14; p = 0.027). In contrast, neither increased cleaning frequency nor the use of antiseptics showed a statistically significant effect on MRCoPS occurrence (Table 2).

Of the 62 MRCoPS isolates, 26 were recovered from environmental surfaces, 5 from canine patients, and 1 from a veterinary staff member. Floor samples accounted for the majority of environmental contamination (16/26; 61.54%), followed by examination tables and other high-contact surfaces. The Dermatology and Post-surgery Care Units exhibited the highest proportion of MRCoPS-positive surfaces (up to 60%), whereas no MRCoPS was detected in the Vaccination Unit (Table 3).

**Table 3:** Distribution of methicillin-susceptible coagulase-positive *Staphylococci* (MSCoPS) and methicillin-resistant coagulase-positive *Staphylococci* (MRCoPS) detected on the floor and on medical instruments^1^.

Unit	Floor	Examination table	Syringe tray	Keyboard	Wound cotton	Doorknob
Gynecology	△	△▲			△◯	
Medicine	△▲◯		△▲	⚫		
Post-operative Care	△	△		△▲		△
Surgery		▲				
Dermatological	△	△	△			△
Vaccine	▲					
ICU	⚫	◯▲				
Hallways	▲⚫					

Gray = Non-detectable, ▲ = Methicillin-susceptible Staphylococcus *pseudintermedius*, △ = Methicillin-resistant *Staphylococcus pseudintermedius*, ⚫ = Methicillin-susceptible *Staphylococcus coagulans*, ◯ = Methicillin-resistant Staphylococcus coagulans.

1 = The data show only instruments from which MSCoPS and MRCoPS were isolated. ICU = Intensive care unit.

**Figure 2:**
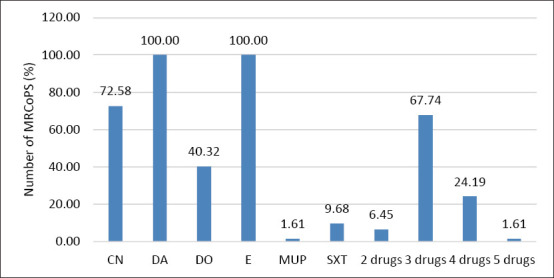
Percentile of antibiotic drugs resistant to clindamycin, doxycycline, erythromycin, gentamicin, mupirocin, and sulfamethoxazole/trimethoprim; 2 drugs = resistant to 2 antibiotic drugs in the same samples; 3 drugs = resistant to 3 antibiotic drugs in the same samples; 4 drugs = resistant to 4 antibiotic drugs in the same samples; 5 drugs = resistant to 5 antibiotic drugs in the same samples.

Seven distinct antibiogram patterns were identified. The predominant resistance phenotype among MRSP isolates was CN–DA–E (CN, DA, and E). All MRSC isolates demonstrated the same triple-resistance pattern (CN–DA–E), confirming a consistent MDR phenotype across species (Figure 2).

### Antimicrobial resistance profiles

All 62 MRCoPS isolates were tested for antimicrobial susceptibility to six commonly used veterinary antibiotics. Universal resistance to DA and E was observed. A high proportion of isolates (58/62; 93.5%) exhibited MDR, defined as resistance to three or more antimicrobial classes.

### SCC*mec* typing

**Figure 3:**
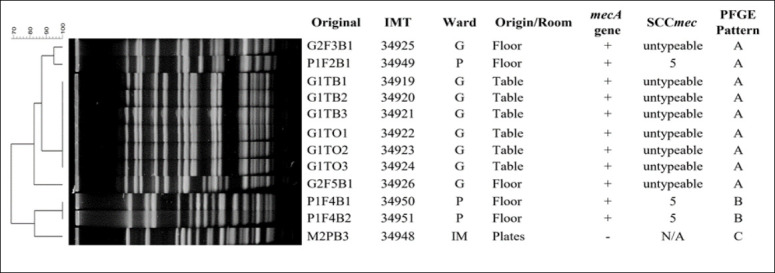
*Staphylococcus pseudintermedius* digested with Apal: The figure shows the staphylococcal cassette *mec* type and pulsed-field gel electrophoresis pattern of *S. pseudintermedius* isolates from various sources in the Small Animal Teaching Hospital; G = Gynecology room, IM = Internal Medicine room, P = Post-surgery, and N/A = not detected.

**Figure 4:**
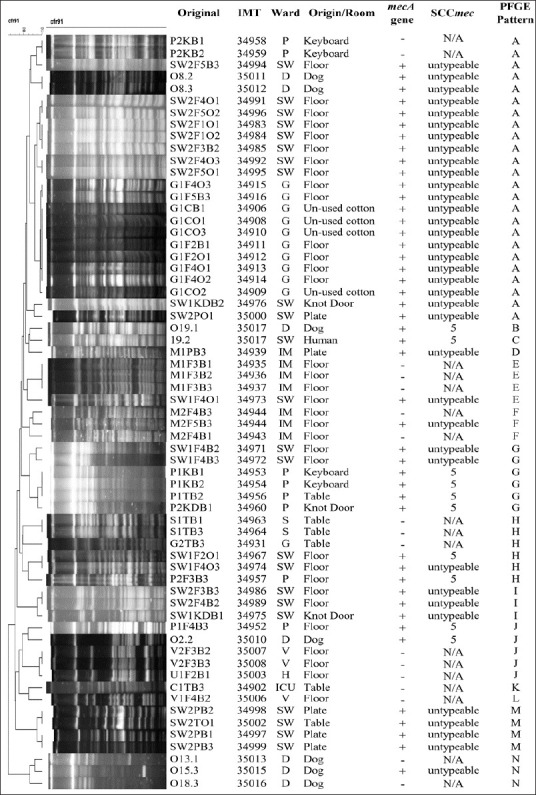
*Staphylococcus pseudintermedius* digested with Cfr9I: The figure shows the staphylococcal cassette *mec* type and pulsed-field gel electrophoresis pattern of *S. pseudintermedius* isolates from various sources in the Small Animal Teaching Hospital; G = Gynecology room, IM = Internal Medicine room, ICU = Intensive care unit, SW = Dermatological room, P = Post-surgery room, S = Surgery room, V = Vaccination room, H = Hall way, D = Dog, and N/A = not detected.

**Figure 5:**
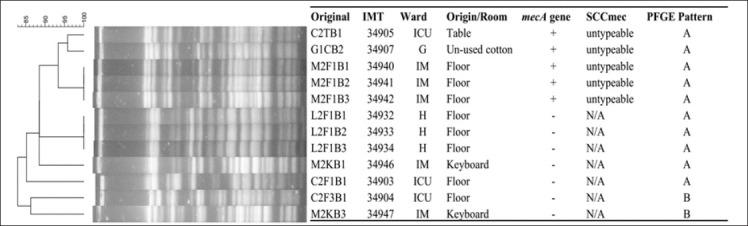
*Staphylococcus coagulans* digested with cfr9I: The figure shows the staphylococcal cassette *mec* type and pulsed-field gel electrophoresis patterns of *S. coagulans* isolates from various sources in the Small Animal Teaching Hospital; G = Gynecology room, IM = Internal Medicine room, ICU = intensive care unit, and H = Hallway.

SCC*mec* typing revealed that all MRSC isolates possessed type V cassettes. In contrast, MRSP isolates exhibited both SCC*mec* type V and several untypeable variants, suggesting the circulation of atypical or possibly novel *mec* element configurations. The heterogeneity of SCC*mec* types among MRSP correlated with their diverse antimicrobial resistance profiles (Figures 3–5).

### Clonal relatedness of isolates

PFGE analysis demonstrated considerable genetic diversity among MRCoPS isolates. *S. pseudintermedius* isolates were grouped into 14 distinct PFGE patterns (A–N), while *S. coagulans* isolates were categorized into two major patterns (A and B). Notably, PFGE pattern A was shared among MRSP isolates from dogs and environmental samples obtained from the Dermatology, Gynecology, and Post-surgery Care units. This finding strongly indicates clonal dissemination of MRSP strains within the hospital environment.

Twelve *S. pseudintermedius* isolates were non-typeable using Cfr9I digestion but were successfully resolved with ApaI, yielding three additional unique banding profiles. This result suggests the presence of methylated or atypical restriction sites in certain isolates, reflecting genetic variability and potential genomic adaptation within circulating MRSP strains.

## DISCUSSION

The management and veterinary staff at the VTH-CU, as in other major veterinary hospitals worldwide, recognize the critical threat posed by nosocomial infections, particularly those involving MDR pathogens such as MRCoPS. This study was therefore designed to systematically investigate the presence and distribution of CoPS and MRCoPS within the hospital environment, encompassing medical instruments, equipment, floors, staff nasal cavities, and lesions from canine patients. Identifying contamination “hotspots” for these pathogens is essential for guiding the implementation of improved infection prevention and control strategies in the VTH-CU.

### Environmental persistence and reservoirs

MRCoPS, particularly MRSP, were frequently isolated from hospital floors and medical instruments across multiple rooms, indicating substantial environmental persistence despite routine sanitation. The consistently high contamination rate on floor surfaces, especially in the Dermatological Unit, reveals gaps in existing cleaning and disinfection protocols. Floors often act as reservoirs for pathogen accumulation and serve as hubs for indirect transmission through human footwear, equipment, or animal contact.

Although the VTH-CU followed standard cleaning procedures, the persistence of MRCoPS suggests potential inefficiencies related to disinfectant type, concentration, or application technique. More effective agents, such as sodium hypochlorite, 2% phenolic solutions, or 0.5% chlorhexidine, should be considered for integration into hospital sanitation regimens [25, 39]. In cases of persistent contamination, advanced surface-decontamination methods such as levulinic acid–sodium dodecyl sulfate combinations or the “MoWa” (spray-wash) technique have shown promising results [40]. Ensuring sufficient disinfectant contact time (≥20 min) and replacing mop-bucket solutions at least twice daily are critical operational measures. The persistence of MRCoPS even after cleaning underscores the need to revise these protocols as part of a targeted hospital-wide infection control plan.

### Disinfection practices and protocol optimization

Examination tables, although disinfected between patient uses, were also found to harbor residual MRSP, particularly in the Dermatology, Gynecology, ICU, and Post-surgery Care Units. This persistence suggests suboptimal disinfection, likely due to inadequate contact duration or the use of less potent agents. Evidence-based cleaning guidelines, such as those recommended by Portner and Johnson [25], should be strictly followed.

In addition, reinforcing World Health Organization (WHO) hand-hygiene standards (e.g., alcohol-based rubs or 0.5% chlorhexidine) and adopting rapid-action disinfectants such as hydrogen peroxide, peracetic acid, or peroxymonosulfate, which provide significant microbial reduction within 5 min, could substantially reduce surface contamination and cross-transmission risks [41].

### Implications of antimicrobial resistance

The widespread resistance of MRCoPS to critical antibiotics, notably DA and E, reflects an alarming MDR phenotype. This trend aligns with global evidence linking extensive antibiotic use, especially β-lactams, to the emergence of resistance in pathogens such as *Enterococcus faecium*, *Acinetobacter baumannii*, and *Pseudomonas aeruginosa* [42]. At the VTH-CU, β-lactam antibiotics were the most commonly prescribed in three clinical areas, yet MRCoPS were detected in five units, including one where enrofloxacin was predominantly used. Given that most MRCoPS isolates displayed resistance to multiple antibiotic classes, the routine empirical use of β-lactams may be ineffective against circulating strains. This highlights the urgent need to implement a robust Antimicrobial Stewardship Program (ASP) and continuous resistance surveillance to guide rational antibiotic use and minimize the spread of resistant pathogens [43, 44]. Although MRSA has been the traditional focus of most veterinary hospital studies, the present findings emphasize the growing clinical relevance of MRSP. Interestingly, reports from Brazil have documented a higher prevalence of MRSA than MRSP [43], whereas the current data from Thailand indicate the opposite. These differences may reflect geographical variation, antibiotic usage patterns, and local infection-control practices [13, 22].

### MRSP prevalence in Thai veterinary facilities

The higher prevalence of MRSP observed in this study compared with previous reports from veterinary hospitals in southern Thailand [19, 22] can be attributed to the scale and activity level of the VTH-CU. As a central referral hospital managing a significantly larger patient load, the VTH-CU presents increased opportunities for bacterial shedding and environmental contamination. High patient turnover and the diverse range of clinical services contribute to the persistence and dissemination of MRSP across multiple hospital units.

### Occupational exposure and human health risks

An MDR MRSP isolate was recovered from the nasal cavity of one veterinarian, consistent with previous findings of occupational exposure in veterinary personnel. Although MRSP carriage in humans remains relatively uncommon, it is strongly associated with close animal contact in veterinary settings [15, 29, 45, 46]. The reported human MRSP carriage rate typically ranges from 1% to 5%, with Thailand showing the highest prevalence (8%) [29].

In this study, the PFGE profile of the human isolate differed from those of canine or environmental sources, suggesting independent acquisition rather than direct transmission. Nonetheless, this underscores the occupational hazard faced by veterinary professionals. Stringent hand hygiene, proper use of personal protective equipment (PPE), and adherence to WHO protocols are essential preventive strategies, particularly in high-risk areas [26, 47–49].

### Clonal dissemination and genetic diversity

The frequent use of antimicrobials in veterinary hospitals promotes the emergence of resistant commensal bacteria on animal skin and mucosal surfaces [50]. All canine patients in this study harbored CoPS, though only a subset carried methicillin resistance. PFGE analysis revealed a shared clonal pattern (pattern A) among MRSP isolates from dogs and environmental surfaces in the Dermatology, Gynecology, and Post-surgery Care Units, indicating clonal dissemination within the hospital. This pattern aligns with earlier findings of MRSP contamination on surgical instruments and in infected dogs [19].

Some MRSP isolates were untypeable by standard SCC*mec* typing, possibly due to atypical or hybrid genetic elements such as ΨSCC*mec*57395 or SCC*mec*AI16-SCCczrAI16-CI, previously detected in Thailand [50, 51]. The diversity of SCC*mec* structures and their association with MDR highlight the dynamic genetic evolution of MRSP under selective antimicrobial pressure. Given that SCC*mec* is a mobile element capable of transferring resistance genes across Staphylococcus species, stringent infection-control policies are critical to curbing its intra-hospital dissemination.

Interestingly, 12 *S. pseudintermedius* isolates were non-typeable using Cfr9I but could be digested with ApaI, a restriction enzyme used for MRSA sequence type 398 [52]. This difference implies the presence of methylated or atypical restriction sites and further underscores the genetic heterogeneity of circulating MRSP strains. Whole-genome sequencing is warranted to characterize these untypeable isolates and elucidate potential novel genomic adaptations.

### Future directions and one health implications

The findings reaffirm the interconnectedness of animal, human, and environmental health within the One Health framework. MRCoPS, including MRSA, MRSP, and MRSC, are zoonotic agents capable of cross-transmission between species and persistence on environmental surfaces and medical devices [13, 18, 53, 54]. The detection of identical or closely related clones among hospital staff, patients, and surfaces underscores the need for integrated surveillance and infection-control systems.

Based on these insights, a comprehensive infection-control strategy for the VTH-CU is recommended, structured around three domains:

1. Human domain: Enforce strict hand hygiene (WHO’s five moments of hand hygiene [55]), PPE usage (gloves, masks, and gowns), and occupational screening programs to minimize zoonotic exposure [26, 47, 48]
2. Animal domain: Implement prudent antimicrobial use, targeted screening for high-risk or recurrently treated animals, and isolation protocols for colonized or infected patients
3. Environmental domain: Strengthen cleaning frequency (especially in high-risk units), ensure appropriate disinfectant selection and contact time, and establish standardized sanitation and accreditation frameworks [22, 43].

Annual environmental and clinical MRCoPS surveillance should be institutionalized to support early detection and continuous quality improvement. Collectively, these interventions will mitigate the spread of MDR Staphylococcus species, safeguard both animal and human health, and reinforce biosecurity within veterinary facilities.

## CONCLUSION

This study provides comprehensive insight into the prevalence, molecular epidemiology, and antimicrobial resistance of MRCoPS in a major VTH in Thailand. Among 253 samples collected from the environment, veterinary personnel, and canine patients, *S. pseudintermedius* predominated (85.23%), followed by *S. coagulans* (13.64%), with 70.5% of all isolates confirmed as methicillin-resistant. Floors and high-contact surfaces, particularly in the Dermatological and Post-surgery Care Units, represented major environmental reservoirs, while one veterinary staff member harbored an MDR MRSP strain. Most MRCoPS isolates were resistant to DA and E, and 93.5% exhibited MDR, highlighting the threat of therapeutic failure and nosocomial transmission. PFGE analysis confirmed clonal relatedness between canine and environmental isolates, indicating intra-hospital dissemination.

These findings emphasize the urgent need for strengthened hospital sanitation protocols, evidence-based disinfectant use, and routine environmental surveillance. Implementation of ASPs, combined with hand hygiene compliance and PPE use, can substantially reduce the risk of MRCoPS transmission among animals and staff. The study’s main strength lies in its integrated approach, linking molecular typing, antimicrobial profiling, and statistical risk assessment. However, it was limited to a single institution and a cross-sectional sampling design, which restricts temporal interpretation and generalization to other hospital settings.

Longitudinal monitoring, genomic characterization of untypeable SCC*mec* variants, and evaluation of disinfection efficacy are warranted to better understand persistence mechanisms and emerging clones. The detection of widespread MRSP contamination and evidence of clonal spread underscore the necessity of adopting One Health-based infection-control strategies to safeguard both animal and human health within veterinary facilities.

## AUTHORS’ CONTRIBUTIONS

PF: Designed the study, sampling, isolation, characterization of the various bacterial isolates, pulsed-field gel electrophoresis, data analysis, and drafted the manuscript. JM: Conducted MALDI-TOF MS, analyzed the results, and edited the manuscript. DJH and NP: Conceptualized the study, analyzed the data, and edited the manuscript. All authors have read and approved the published version of the manuscript.

## References

[1] Yang C, Chen X, Li M, Yuan W, Li S, Han D, Feng J, Luo H, Zheng M, Liang J, Chen C, Qu P, Li S (2025). Author correction:Genomic epidemiology and phenotypic characterization of *Staphylococcus aureus* isolated from atopic dermatitis patients in South China. Sci. Rep.

[2] Pleskova S.N, Bobyk S.Z, Kriukov R.N, Gorshkova E.N, Bezrukov N.A (2022). *Staphylococcus aureus* causes the arrest of neutrophils in the bloodstream in a septicemia model. Microorganisms.

[3] Ariens R.A.S, Cassat J.E (2023). Surviving a sticky situation:Therapeutic administration of fibrinogen variant γ'improves outcomes of *Staphylococcus aureus* septicemia. J. Thromb. Haemost.

[4] Marek L, Irimaso E, Turikumwenayo J.B, Mukamulisa B, Ndishimye P, Muragijemariya F, Cabal-Rosel A, Desvars-Larrive A, Fischer O.W, Szostak M.P, Muller E, Braun S.D, Ehling-Schulz M, Spergser J, Grunert T, Ruppitsch W, Fessler A.T, Schwarz S, Monecke S, Ehricht R, Kunzel F, Loncaric I (2025). *Staphylococcus aureus* in Rwandan dogs predominantly representing human-associated lineages. Lett. Appl. Microbiol.

[5] Katakweba A.A.S, Iversen C.M, Tsaxra J.B, Muhairwa A.P, Moodley A, Olsen J.E (2024). Brief communication:Carrier rate, antimicrobial resistance and molecular typing of *Staphylococcus aureus* and *Staphylococcus pseudintermedius* in healthy dogs from Morogoro, Tanzania. Vet. Dermatol.

[6] Del Pilar Zarazaga M, Tinti M.G, Litterio N.J, Himelfarb M.A, Andres-Larrea M.I.S, Rubio-Langre S, Serrano-Rodriguez J.M, Lorenzutti A.M (2024). Dose regimen optimization of cephalothin for surgical prophylaxis against *Staphylococcus aureus* and coagulase-negative staphylococci in dogs by pharmacokinetic/pharmacodynamic modeling. Res. Vet. Sci.

[7] Cengiz S, Okur S, Oz C, Turgut F, Gumurcinler B, Sevuk N.S, Kekec A.I, Cepoglu H, Sevimli U, Adiguzel M.C (2023). Prevalence and clonal diversity of methicillin-resistant *Staphylococcus aureus* and methicillin-resistant *Staphylococcus pseudintermedius* isolated from dogs and cats with eye discharge. Acta Microbiol. Immunol. Hung.

[8] Hammer N.D, Cassat J.E, Noto M.J, Lojek L.J, Chadha A.D, Schmitz J.E, Creech C.B, Skaar E.P (2014). Inter-and intraspecies metabolite exchange promotes virulence of antibiotic-resistant *Staphylococcus aureus*. Cell Host Microbe.

[9] Youssef C.R.B, Kadry A.A, El-Ganiny A.M (2022). Investigating the relation between resistance pattern and type of Staphylococcal cassette chromosome *mec* (SCC*mec*) in methicillin-resistant *Staphylococcus aureus*. Iran J. Microbiol.

[10] Uehara Y (2022). Current status of staphylococcal cassette chromosome *mec* (SCC*mec*). Antibiotics (Basel).

[11] Baranova M.N, Soboleva E.A, Kornienko M.A, Malakhova M.V, Mokrushina Y.A, Gabibov A.G, Terekhov S.S, Smirnov I.V (2024). Bacteriocin from the raccoon dog oral microbiota inhibits the growth of pathogenic methicillin-resistant *Staphylococcus aureus*. Acta Naturae.

[12] Moses I.B, Esimone C.O, Iroha I.R, Rubin J.E, Sniatynsky M.K, Ribeiro A, Santos F.F, Cayo Da Silva R, Gales A.C (2022). Antibiotypes and high frequency of toxin genes in methicillin-resistant *Staphylococcus pseudintermedius* from nares of dogs and dog guardians in Nigeria. Comp. Immunol. Microbiol. Infect. Dis.

[13] Soimala T, Wasiksiri S, Boonchuay K, Wongtawan T, Fungwithaya P (2024). Methicillin-resistant coagulase-positive staphylococci in new, middle-aged, and old veterinary hospitals in Southern Thailand:A preliminary study. Vet World.

[14] Olivo G, Zakia L.S, Ribeiro M.G, Da Cunha M, Riboli D.F.M, Mello P.L, Teixeira N.B, De Araujo C.E.T, Oliveira-Filho J.P, Borges A.S (2024). Methicillin-resistant *Staphylococcus* spp. investigation in hospitalized horses and contacting personnel in a teaching veterinary hospital. J. Equine Vet. Sci.

[15] Kitano H, Kitagawa H, Hisatsune J, Tsunoi M, Kohada Y, Takemoto K, Miyamoto S, Kobatake K, Tadera K, Omori K, Sugai M, Ohge H, Hinata N (2025). First human case of infected lymphocele with bloodstream infection caused by *Staphylococcus coagulans* transmitted from a pet dog:A case report. Diagn. Microbiol. Infect. Dis.

[16] Denu R.A, Patel D, Becker B.J, Shiffler T, Kleinschmidt P (2020). MRSA septicemia with septic arthritis and prostatic, intraretinal, periapical, and lung abscesses. Wis. Med. J.

[17] Fungwithaya P, Chanchaithong P, Phumthanakorn N, Muaungkong P, Bumpenpol P, Kaewparuehaschai M, Tribuddharat C, Prapasarakul N (2016). Association between cephalexin administration and emergence of methicillin-resistant coagulase-positive staphylococci (MRCoPS) in dogs. Thai J. Vet. Med.

[18] Afshar M.F, Zakaria Z, Cheng C.H, Ahmad N.I (2023). Prevalence and multidrug-resistant profile of methicillin-resistant *Staphylococcus aureus* and methicillin-resistant *Staphylococcus pseudintermedius* in dogs, cats, and pet owners in Malaysia. Vet. World.

[19] Fungwithaya P, Brikshavana P, Chanchaithong P, Prapasarakul N (2017). Distribution of methicillin-resistant coagulase-positive staphylococci (MRCoPS) in a surgical unit and cystotomy operation sites in a veterinary teaching hospital. J. Vet. Med. Sci.

[20] Bergstrom A, Gustafsson C, Leander M, Fredriksson M, Gronlund U, Trowald-Wigh G (2012). Occurrence of methicillin-resistant Staphylococci in surgically treated dogs and the environment in a Swedish animal hospital. J. Small Anim. Pract.

[21] Bergstrom K, Aspán A, Landén A, Johnston C, Grönlund U (2012). The first nosocomial outbreak of methicillin-resistant *Staphylococcus aureus* in horses in Sweden. Acta Vet. Scand.

[22] Abusleme F, Galarce N, Quezada-Aguiluz M, Iraguen D, Gonzalez-Rocha G (2022). Characterization and antimicrobial susceptibility of coagulase-positive *Staphylococcus* isolated in a veterinary teaching hospital in Chile. Rev. Argent Microbiol.

[23] Hunter N.D, Hoet A.E, Van Balen J, Stull J.W (2021). Longitudinal environmental *Staphylococcus* contamination in a new small animal veterinary hospital and utility of cleaning checklists. Zoonoses Public Health.

[24] Fungwithaya P, Sontigun N, Boonhoh W, Boonchuay K, Wongtawan T (2022). Antimicrobial resistance in *Staphylococcus pseudintermedius* on the environmental surfaces of a recently constructed veterinary hospital in Southern Thailand. Vet. World.

[25] Portner J.A, Johnson J.A (2010). Guidelines for reducing pathogens in veterinary hospitals:Disinfectant selection, cleaning protocols, and hand hygiene. Compend. Contin. Educ. Vet.

[26] Schmitt K, Zimmermann A.B.E, Stephan R, Willi B (2021). Hand hygiene evaluation using two different evaluation tools and hand contamination of veterinary healthcare workers in a swiss companion animal clinic. Vet. Sci.

[27] Patchanee P, Tadee P, Ingkaninan P, Tankaew P, Hoet A.E, Chupia V (2014). Distribution and characterization of methicillin-resistant *Staphylococcus aureus* (MRSA) at the small animal hospital, faculty of veterinary medicine, Chiang Mai University, Thailand. Southeast Asian J. Trop. Med. Public. Health.

[28] Buttner M.P, Cruz-Perez P, Stetzenbach L.D (2001). Enhanced detection of surface-associated bacteria in indoor environments by quantitative PCR. Appl. Environ. Microbiol.

[29] Chanchaithong P, Perreten V, Schwendener S, Tribuddharat C, Chongthaleong A, Niyomtham W, Prapasarakul N (2014). Strain typing and antimicrobial susceptibility of methicillin-resistant coagulase-positive staphylococcal species in dogs and people associated with dogs in Thailand. J. Appl. Microbiol.

[30] Chanchaithong P, Prapasarakul N (2011). Biochemical markers and protein pattern analysis for canine coagulase-positive staphylococci and their distribution on dog skin. J. Microbiol. Methods.

[31] Sasaki T, Tsubakishita S, Tanaka Y, Sakusabe A, Ohtsuka M, Hirotaki S, Kawakami T, Fukata T, Hiramatsu K (2010). Multiplex-PCR method for species identification of coagulase-positive staphylococci. J. Clin. Microbiol.

[32] Murugaiyan J, Walther B, Stamm I, Abou-Elnaga Y, Brueggemann-Schwarze S, Vincze S, Wieler L.H, Lubke-Becker A, Semmler T, Roesler U (2014). Species differentiation within the *Staphylococcus intermedius* group using a refined MALDI-TOF MS database. Clin. Microbiol. Infect.

[33] CLSI (2013). Performance Standards for Antimicrobial Disk and Dilution Susceptibility Tests for Bacteria Isolated from Animals;Second Informational Supplement (Document VET01-S2). Clinical and Laboratory Standards Institute, Wayne, .26..

[34] Strommenger B, Kettlitz C, Werner G, Witte W (2003). Multiplex PCR assay for simultaneous detection of nine clinically relevant antibiotic resistance genes in *Staphylococcus aureus*. J. Clin. Microbiol.

[35] Maaland M.G, Papich M.G, Turnidge J, Guardabassi L (2013). Pharmacodynamics of doxycycline and tetracycline against *Staphylococcus pseudintermedius*:Proposal of canine-specific breakpoints for doxycycline. J. Clin. Microbiol.

[36] Kondo Y, Ito T, Ma X.X, Watanabe S, Kreiswirth B.N, Etienne J, Hiramatsu K (2007). Combination of multiplex PCRs for staphylococcal cassette chromosome *mec* type assignment:Rapid identification system for *mec*, *ccr*, and major differences in junkyard regions. Antimicrob. *Agents Chemother*.

[37] Bergstrom K, Nyman G, Widgren S, Johnston C, Gronlund-Andersson U, Ransjo U (2012). Infection prevention and control interventions in the first outbreak of methicillin-resistant *Staphylococcus aureus* infections in an equine hospital in Sweden. Acta Vet. Scand.

[38] Murchan S, Kaufmann M.E, Deplano A, De Ryck R, Struelens M, Zinn C.E, Fussing V, Salmenlinna S, Vuopio-Varkila J, El Solh N, Cuny C, Witte W, Tassios P.T, Legakis N, Van Leeuwen W, Van Belkum A, Vindel A, Laconcha I, Garaizar J, Haeggman S, Olsson-Liljequist B, Ransjo U, Coombes G, Cookson B (2003). Harmonization of pulsed-field gel electrophoresis protocols for epidemiological typing of strains of methicillin-resistant *Staphylococcus aureus*:A single approach developed by consensus in 10 European laboratories and its application for tracing the spread of related strains. J. Clin. Microbiol.

[39] Umemura T, Mutoh Y, Maeda M, Hagihara M, Ohta A, Mizuno T, Kato H, Sukawa M, Yamada T, Ikeda Y, Mikamo H, Ichihara T (2022). Impact of hospital environmental cleaning with a potassium peroxymonosulphate-based environmental disinfectant and antimicrobial stewardship on the reduction of hospital-onset Clostridioides difficile infections. J. Hosp. Infect.

[40] Gregory T.V, Ellis K, Valeriani R, Khan F, Wu X, Murin L, Alibayov B, Vidal A.G.J, Zhao T, Vidal J.E (2021). MoWa:A disinfectant for hospital surfaces contaminated with methicillin-resistant *Staphylococcus aureus* (MRSA) and other nosocomial pathogens. Front Cell Infect. *Microbiol*.

[41] Ling M.L, How K.B (2012). Impact of a hospital-wide hand hygiene promotion strategy on healthcare-associated infections. Antimicrob. Resist. Infect. Control.

[42] Abejew A.A, Wubetu G.Y, Fenta T.G (2024). Relationship between antibiotic consumption and resistance:A systematic review. Can. J. Infect. Dis. Med. Microbiol.

[43] Samarkos M (2024). Antimicrobial stewardship program (ASP) in a general hospital:An essential practice. Antibiotics (Basel).

[44] Wang X, Lin L, Xu X, Harbarth S, Yakob L, Zhang R, Zhou X (2025). Implementing the Chinese mandatory antimicrobial stewardship program:Barriers to continuous improvement. Health Policy Plan.

[45] Blondeau L.D, Deutscher M, Rubin J.E, Deneer H, Kanthan R, Sanche S, Blondeau J.M (2022). Urinary tract infection in a human male patient with *Staphylococcus pseudintermedius* transmission from the family dog. J. Chemother.

[46] Nomoto H, Kutsuna S, Nakamura K, Nakamoto T, Shimomura A, Hirakawa T, Kinoshita N, Hayakawa K, Nagashima M, Ohmagari N (2020). Totally implantable venous access port infection caused by *Staphylococcus pseudintermedius*:Possible transmission from a companion dog to a human. J. Infect. Chemother.

[47] Kelly S, Winzor G, Lam S.C (2025). World Hand Hygiene Day 2025:A perspective from the infection prevention in practice editors. Infect. Prev. Pract.

[48] Lotfinejad N, Peters A, Tartari E, Fankhauser-Rodriguez C, Pires D, Pittet D (2021). Hand hygiene in health care:20 years of ongoing advances and perspectives. Lancet Infect. Dis.

[49] CDC (2025). The Basics of Standard Precautions. https://www.cdc.gov/infection-control/media/pdfs/strive-pp101-508.pdf.

[50] Perreten V, Chanchaithong P, Prapasarakul N, Rossano A, Blum S.E, Elad D, Schwendener S (2013). Novel pseudo-staphylococcal cassette chromosome *mec* element (psiSCC*mec*57395) in methicillin-resistant *Staphylococcus pseudintermedius* CC45. Antimicrob. Agents Chemother.

[51] Chanchaithong P, Prapasarakul N, Perreten V, Schwendener S (2016). Characterization of a novel composite staphylococcal cassette chromosome *mec* in methicillin-resistant *Staphylococcus pseudintermedius* from Thailand. Antimicrob. Agents Chemother.

[52] Rasschaert G, Vanderhaeghen W, Dewaele I, Janez N, Huijsdens X, Butaye P, Heyndrickx M (2009). Comparison of fingerprinting methods for typing methicillin-resistant *Staphylococcus aureus* sequence type 398. J.Clin. Microbiol.

[53] Frosini S.M, Bond R, King R.H, Loeffler A (2022). The nose is not enough:Multi-site sampling is best for MRSP detection in dogs and households. Vet. Dermatol.

[54] Dodds W.J (2025). One health:Introduction to integrative veterinary medicine. *Vet*. *Clin*. *North Am*. *Small Anim*. Pract.

[55] WHO (2025). Your 5 Moments for Hand Hygiene. https://cdn.who.int/media/docs/default-source/integrated-health-services-(ihs)/infection-prevention-and-control/your-5-moments-for-hand-hygiene-poster.pdf..

